# Perinatal Protein Malnutrition Affects Mitochondrial Function in Adult and Results in a Resistance to High Fat Diet-Induced Obesity

**DOI:** 10.1371/journal.pone.0104896

**Published:** 2014-08-13

**Authors:** Céline Jousse, Yuki Muranishi, Laurent Parry, Christophe Montaurier, Patrick Even, Jean-Marie Launay, Valérie Carraro, Anne-Catherine Maurin, Julien Averous, Cédric Chaveroux, Alain Bruhat, Jacques Mallet, Béatrice Morio, Pierre Fafournoux

**Affiliations:** 1 UMR1019 Nutrition Humaine, Institut National de la Recherche Agronomique (INRA), Université Clermont 1, Saint Genès Champanelle, France; 2 Institut National de la Recherche Agronomique (INRA), AgroParisTech, UMR914 Nutrition Physiology and Ingestive Behavior, Paris, France; 3 Service de Biochimie et Biologie Moléculaire/Equipe Associée (EA) 3621, Assistance Publique des Hôpitaux de Paris (AP-HP), Hôpital Lariboisière, Faculté de Pharmacie, Université Paris Descartes, Paris, France; 4 Laboratoire de Génétique Moléculaire de la Neurotransmission et des Processus Neurodégénératifs, UMR7091, Université Pierre et Marie Curie (UPMC) Paris Université/CNRS, Hôpital de la Pitié-Salpêtrière, Paris, France; INRA, France

## Abstract

Epidemiological findings indicate that transient environmental influences during perinatal life, especially nutrition, may have deleterious heritable health effects lasting for the entire life. Indeed, the fetal organism develops specific adaptations that permanently change its physiology/metabolism and that persist even in the absence of the stimulus that initiated them. This process is termed “nutritional programming”. We previously demonstrated that mothers fed a Low-Protein-Diet (LPD) during gestation and lactation give birth to F1-LPD animals presenting metabolic consequences that are different from those observed when the nutritional stress is applied during gestation only. Compared to control mice, adult F1-LPD animals have a lower body weight and exhibit a higher food intake suggesting that maternal protein under-nutrition during gestation and lactation affects the energy metabolism of F1-LPD offspring. In this study, we investigated the origin of this apparent energy wasting process in F1-LPD and demonstrated that minimal energy expenditure is increased, due to both an increased mitochondrial function in skeletal muscle and an increased mitochondrial density in White Adipose Tissue. Importantly, F1-LPD mice are protected against high-fat-diet-induced obesity. Clearly, different paradigms of exposure to malnutrition may be associated with differences in energy expenditure, food intake, weight and different susceptibilities to various symptoms associated with metabolic syndrome. Taken together these results demonstrate that intra-uterine environment is a major contributor to the future of individuals and disturbance at a critical period of development may compromise their health. Consequently, understanding the molecular mechanisms may give access to useful knowledge regarding the onset of metabolic diseases.

## Introduction

Metabolic diseases, including obesity and Type II Diabetes Mellitus (T2DM) are among the leading causes of disability in industrialized countries. These diseases have multifactorial causes that involve genetic and environmental factors and they often represent the tip of an iceberg of composite syndromes. Extensive epidemiological findings indicate that a key feature characteristic of these diseases is that transient environmental influences during perinatal life may have deleterious heritable health effects lasting for the entire life [1]. Among these environmental factors, nutrition plays a major role especially during critical windows of development. Indeed, the fetal organism is able to respond to nutritional stresses by specific adaptations at the cellular and molecular levels that permanently change the physiology and the metabolism of the organism and persist even in the absence of the stress/stimulus that initiated them. This process is termed “nutritional programming”.

There is a large number of well-established animal models that indicates a link between perinatal growth and phenotype in the adulthood. Currently, the most widely used animal model of nutritional programming is maternal under-nutrition. For example, female rats fed a Low Protein Diet (LPD) during gestation give birth to pups that exhibit a lower weight at birth but that catch up quickly during the early days of life. In their adulthood, they will be prone to develop glucose intolerance, especially when fed a high fat diet after weaning [2]. These adverse adult health outcomes are associated with permanent changes in the expression of genes involved in glucose homeostasis [3], [4]. In that case, they display a higher vulnerability to obesity and diabetes reflecting an inappropriate match between metabolic programming by the diet received during fetal life and the diet actually consumed after birth.

In a previous work, we reported that perinatal under-nutrition during both gestation and lactation affects the extent of methylation of the leptin promoter in adults and that this methylation is correlated with changes in leptin regulation [5]. However, regarding metabolic parameters, our findings are at variance with those described above highlighting the importance of the time window during which the maternal nutritional stress is applied. Indeed, our data demonstrated that maternal undernutrition during gestation and lactation (F1-LPD animals) leads to metabolic consequences later in adulthood that are different from those observed when nutritional stress is applied during gestation only. Compared to control mice, F1-LPD animals have a lower body weight and exhibit a higher food intake suggesting that maternal protein under-nutrition during gestation and lactation affects the energy metabolism of F1-LPD offspring. In the present study, we investigated the origin of this apparent energy wasting process in F1-LPD mice and demonstrated that minimal energy expenditure is increased in F1-LPD animals, due to both an increased mitochondrial function in skeletal muscle and an increased mitochondrial density in White Adipose Tissue. Importantly, once adult, these mice are protected against high fat diet-induced obesity.

## Methods and Materials

### Ethics Statement

Maintenance of the mice and all experiments were conducted according to the guidelines formulated by the European Community for the use of experimental animals (L358-86/609/EEc) and were approved by the Institut National de la Recherche Agronomique (INRA, France). INRA animal facilities were approved by the French veterinary department (C634514).

### Animal protocols

Balb/c mice (obtained from Charles River Laboratory) were used, and all animals were individually housed in plastic cages and subjected to a 12 h light/dark cycle at a temperature of 22°C (except otherwise indicated). All animals had ad libitum access to food and water at all times, unless otherwise indicated.

### Experimental procedure

Twenty virgin 4-month old female Balb/c mice were used. Pairs of female mice were mated with a single male and, at plug date, were allocated into 2 groups fed either a diet containing 22% of protein (control diet; CD) or a diet containing 10% of protein (low protein diet; LPD) throughout gestation and lactation. Only litters of four to eight pups were included in subsequent experiments. At weaning at 4 weeks of age, the male offspring (called F1-CD and F1-LPD) were single-housed and were given standard chow diet (A03; SAFE) throughout life. The diets compositions have been previously described [5]. For each experiment, at least 6 representative males from at least 4 different litters were selected in each group.

### High Fat diet challenge procedure

When indicated, at 5 months of age and for each experimental group, 8 male mice were switched to High Fat Diet (HFD) (D12451, Research Diet containing 24% Protein, 41% Carbohydrates, 24% Fat), and 8 male mice were fed on Chow diet for 7 months.

### Measurement of food intake

Mice were given ad libitum access to a known amount of pellets for 1 week. At the end of the test, left over food was weighed and the daily amount of food consumed was calculated as the mean intake in grams per day. The food intake is expressed in grams per day per g of Body Weight (BW) or per g of Lean Body Mass (LBM). When indicated, the food intake is expressed in daily Kcal intake calculated according to the nutritional values of each diet (3.2 kCal/g for Chow diet and 4.73 kCal/g for HFD).

### Fat and lean mass determination

The individual mice were placed into restrain tube and inserted into the mouse EchoMRI-100 instrument (Echo Medical Systems LLC, Houston TX, USA) to determine both fat and lean mass (g). Total body weight was measured using a standard top-loading laboratory balance. Adiposity is expressed as a percentage of fat relative to total body weight.

### Measurement of metabolites and hormones

The plasma glucose concentration was measured by using a glucose reagent strip and a glucometer (Glucotouch, Lifescan, Milpitas, CA). Insulin was measured by using an enzyme-linked immunosorbent assay kit purchased from Alpco Diagnostics (Salem, USA). Fasting plasma lipids (ie, triglycerides, total cholesterol and HDL cholesterol) were measured in plasma samples by using an automated system (Konelab 20; ThermoElectron Corporation).

### Indirect calorimetry and spontaneous activity

Dioxygen consumption (VO_2_), carbon dioxide production (VCO_2_) and activity of F1-CD and F1-LPD mice were measured under Lab Chow diet using a four-cage TSE System PhenoMaster/LabMaster (Bad Homburg, Germany). Energy expenditure was calculated using Weir's equation (EE = 16.3 V02+4.57 VCO2) [6] from measurements of gas exchanges computed for each cage from data sampled every 5 min. Spontaneous activity was measured using a three dimensions meshing of light beams. Each cage has a volume of 4.9 l and the flow-rate was set to 0.4 l/min. The O_2_ and CO_2_ analyzer were calibrated before each measurement period. Ambient temperature was maintained at 28°C to reach the animal thermo-neutrality and the light was on from 08am to 08pm. During an adaptation period of twenty-four hours prior to data collection, mice were placed in separate calorimetry cages, with free access to food and water. Then VO_2_, VCO_2_ and activity were monitored during 24 h while fed ad libitum and thereafter, during 16 h while fasting. Daily energy expenditure and activity were computed from the 24 h period when animals were fed. Minimal energy expenditure was calculated as the mean value of the 4 last values at the end of the fasting period. The RQ was calculated as the ratio of VCO_2_ to VO_2_.

### Body Temperature

Body temperature (degrees Celsius) was measured by using a rectal thermo-probe.

### Tissue Collection

At 7 months of age, overnight starved male offspring were killed by pentobarbital overdose, blood was collected by decapitation and various tissues were isolated, frozen in Liquid Nitrogen and stored at −80°C until use. Epididymal white adipose tissue (WAT) was collected from areas surrounding the epididymis and testis, Brown adipose tissue (BAT) was collected from the interscapular region and separated from the attached white adipose tissue, and Gastrocnemius was dissected.

### Measurement of enzyme activities

Maximum activity of complexes II, III and IV (cytochrome c oxidase, COX) was assessed spectrophotometrically on tissue homogenates, as previously described by Barrientos *et al.* [7]. Citrate synthase activity was measured as described by Shepherd [8]. Complex I activity was measured on muscle homogenates using the Complex I Enzyme Activity Dipstick Assay Kit (MitoSciences #MS130). Briefly Complex I is immunocaptured and immuno-precipitated in active form on the dipstick. Secondly, the dipstick is immersed in Complex I activity buffer solution containing NADH as a substrate and nitrotetrazolium blue (NBT) as the electron acceptor. Immunocaptured Complex I oxidizes NADH and the resulting H+ reduces NBT to form a blue-purple precipitate at the Complex I line on the dipstick. The signal intensity of this precipitate corresponds to the level of Complex I enzyme activity in the sample. The signal intensity is analyzed by a standard imaging system.

### Mitochondrial density measurement

Total DNA was extracted from white adipose tissue and from gastrocnemius using phenol/chloroform/isoamyl alcohol (25∶24∶1) followed by ethanol precipitation. The content of mtDNA was calculated using real-time quantitative PCR by measuring the threshold cycle ratio (ΔCt) of a mitochondrial-encoded gene (COX1, forward 5′-ACTATACTACTACTAACAGACCG-3′, reverse 5′-GGTTCTTTTTTTCCGGAGTA-3′) versus a nuclear-encoded gene (cyclophilin A, forward 5′-ACACGCCATAATGGCACTGG-3′, reverse 5′-CAGTCTTGGCAGTGCAGAT-3′).

### Analysis of gene expression using real time RT-PCR

Total RNA was prepared using a RNeasy mini kit (Qiagen) and treated with DNase I, Amp Grade (InVitrogen, Carlsbad, CA, USA) prior to cDNA synthesis. RNA integrity was electrophoretically verified by ethidium bromide staining. RNA (0.5 µg) was reverse transcribed with 100 U of Superscript II plus RNase H- Reverse Transcriptase (InVitrogen) using 100 µM random hexamer primers (Amersham Biosciences, Piscataway, NJ, USA), according to the manufacturer's instructions. Real-time quantitative PCR was carried out on a Bio-Rad CFX-96 detection system with quantitative Q-PCR SYBR Green reagents (Bio-Rad, Hercules, CA, USA) and with a primer concentration of 0.5 µM. PCR conditions were standardized to 40 cycles of 95°C for 10 s and 59°C for 30 s with the primers for specific mouse mRNA sequences (For a list of primer sequences, see Table S1). To control for RNA quality and cDNA synthesis, β-actin or Nono mRNA was also amplified. The abundance of each RNA normalized to the HPRT1 or Nono signal depending on the tissue are expressed as the mean ± SEM of at least 6 samples.

### Protein Analyses by SDS-PAGE Western Blotting

Total proteins were extracted from various tissues using a lysis buffer consisting in 150 mM NaCl, 50 mMTris pH 7,4, 2.5 mM EGTA, 1% Triton X-100, 0.15% CHAPS, 0.5% DOC, 1 mM DTT with the addition of protease and phosphatase inhibitors (Sigma-Aldrich #P8340 and #P0044). Alternatively, for anti-UCP2 western Blotting, proteins were extracted using a High Salt buffer consisting in 25 mM HEPES, 0.4 M NaCl, 1.5 mM MgCl_2_, 0.2 mM EDTA, 1% NP40 with the addition of protease and phosphatase inhibitors (Sigma-Aldrich #P8340 and #P0044). Measurement of protein concentration was performed using the Bradford protein assay kit (BioRad#500-0006). Total protein extracts containing 20 µg of protein were suspended in Laemmli buffer (final concentration: 60 mM Tris-HCl pH 6.8, 2% SDS, 10% glycerol, 0.01% bromophenol blue, 10 mM dTT), and boiled for 5 min. Proteins were separated by electrophoresis and transferred to polyvinylidene fluoride membranes. The membranes were incubated for 1 hour at room temperature with 5% fat-free milk Tris-buffered saline containing 0.1% Tween-20 (TBS-T). The membranes were then incubated overnight at 4°C with the primary antibody diluted 1/1000e in 5% BSA containing TBS-T: anti-CoxIV (Molecular probes #A21348), anti-Porin (Biovision #3594-100), anti-UCP2 (BioLegend #615901), anti-β-Actin (Santa Cruz Biotechnology #sc-47778). After washing in TBS-T, membranes were incubated for 1 hour at room temperature with the secondary antibody diluted 1/5000e in 5% fat-free milk TBS-T (Santa Cruz: goat anti-mouse IgG-HRP sc-2031 for anti-CoxIV and anti-β-Actin and goat anti-rabbit IgG-HRP sc-2030 for anti-Porin and anti-UCP2). After washing in TBS-T, Luminata western HRP substrate (Millipore) and a chemiluminescence imager (G:Box, Syngene) were used to detect the signals.

### Statistical Analysis

Results are reported as mean ± SEM. Unpaired t-tests were used to analyze perinatal undernutrition effect (F1-CD versus F1-LPD). P lower than 0.05 was considered as significant.

## Results

### Maternal malnutrition affects energy expenditure in adult offspring

**Figure 1:**
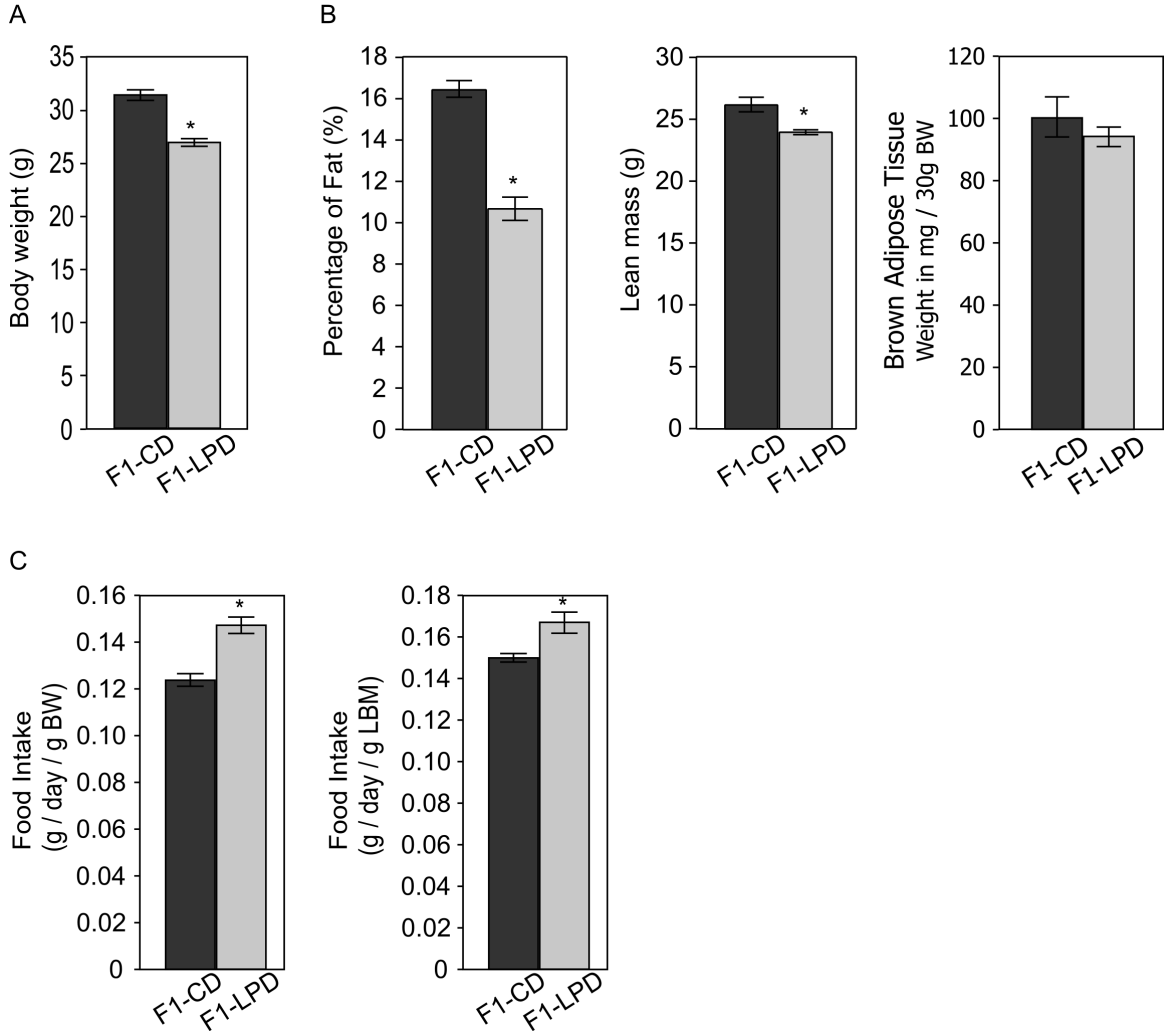
Phenotypic characterization of F1 mice. A) Body weight of 7-mo-old F1-CD and F1-LPD male mice. Results are expressed in grams as means ± sem for at least 10 mice/group. *p≤0.05. B) Body composition parameters of 7-mo-old F1-CD and F1-LPD male mice. Adiposity is expressed as a percentage of fat relative to total body weight. Lean mass is expressed in grams. Brown Adipose Tissue weight is expressed as weight in mg relatively to the weight of each mouse. All values are means ± sem for at least 10 mice/group. *p≤0.05. C) Food intake of 7-mo-old male F1-CD and F1-LPD mice was measured as described in Materials and Methods. Values represent the weight of food consumed daily relatively to the total body weight (BW) or to the Lean Body Mass (LBM) of each mouse and are expressed as means ± sem for at least 10 mice/group. *p≤0.05.

**Figure 2:**
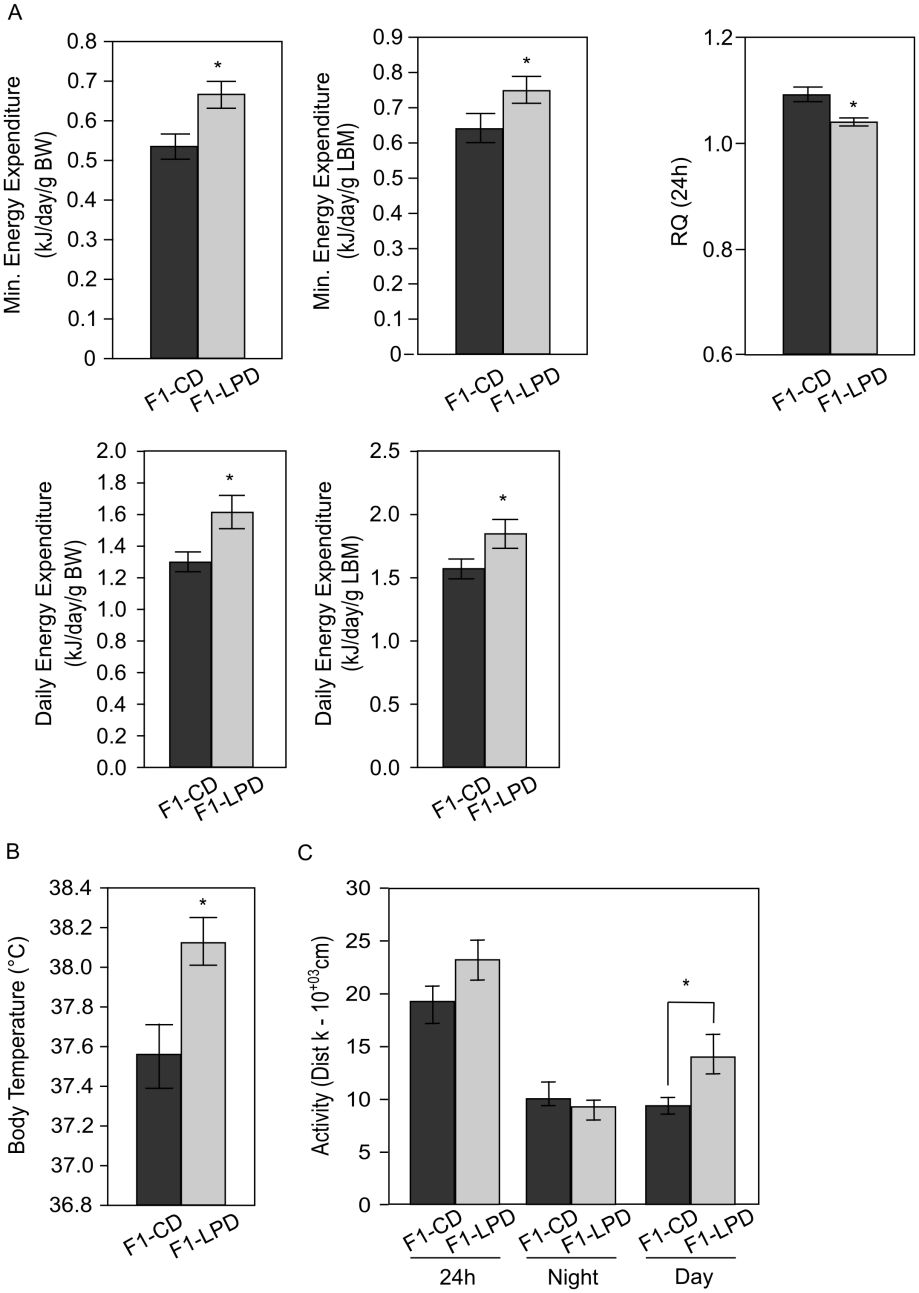
Calorimetric parameters of F1 mice. A) Minimum Energy Expenditure, Daily Energy Expenditure and Respiratory Quotient (RQ) of 7-mo-old male F1-CD and F1-LPD mice were measured by indirect calorimetry as described in Materials and Methods. Energy Expenditure is expressed as kJ per day relatively to the total body weight (BW) or to the Lean Body Mass (LBM) of each mouse. Values are the mean ± sem for 6 mice/group. *p≤0.05. B) Body temperature (°C) of 7-mo-old male F1-CD and F1-LPD mice were measured using a rectal thermoprobe. Values are expressed as the mean temperature ± sem for at least 10 mice/group. *p≤0.05. C) Spontaneous locomotor activity of 7-mo-old male F1-CD and F1-LPD mice were measured in the home cage using infrared light-beam. Values are expressed as the mean distance (10^+03^ cm) ± sem for 6 mice/group. *p≤0.05.

Pregnant and suckling female Balb/c mice were fed either a control diet (CD) or a low protein diet (LPD). After weaning, the F1-CD and F1-LPD male offspring, born respectively to CD- or LPD-fed mothers were fed on a Lab Chow Diet [5]. Figure 1 shows that, once adult, the F1-LPD mice have paradoxical metabolic characteristics. Compared to the F1-CD, adult F1-LPD mice have a lower body weight (Figure 1A), and a lower adiposity and lean mass (Figure1 B) but similar brown adipose tissue weight comparatively to total body weight. However, the food intake of the F1-LPD males is significantly increased compared to F1-CD (Figure 1C) no matter the normalization (total Body weight or Lean Body Mass). Moreover several physiological parameters are affected in F1-LPD mice as detailed in Table 1. Glucose, insulin and triglycerides levels are significantly decreased in plasma of F1-LPD mice. Overall, these results suggest that maternal protein undernutrition during gestation and lactation affects feed efficiency (i.e. the body weight gain per unit of energy consumed) as well as glucose and lipid homeostasis. Therefore, we quantified the components of total energy expenditure in F1-CD and F1-LPD animals by performing indirect calorimetry experiments. Figure 2A shows that both minimal energy expenditure and daily energy expenditure are significantly increased in F1-LPD compared to F1-CD animals, no matter the normalization (total Body weight or Lean Body Mass). Moreover, respiratory quotient (RQ) was lower in F1-LPD compared to F1-CD mice indicating an increased fat oxidation. Energy expenditure referring to the amount of energy used for thermogenesis, physical activity and performance of cellular and organ function, we thus measured body temperature and activity. Figure 2 clearly shows that both rectal temperature (Figure 2B) and activity (Figure 2C) are significantly increased in F1-LPD mice compared to F1-CD animals. It is noticeable that it is the diurnal activity of the mice that is mainly affected.

**Table 1:** Physiological parameters.

	F1-CD	F1-LPD	p-value
Glucose (mg/dL)	89.13±9.07	63.57±8.99	0.026
Insulin (ng/mL)	0.463±0.034	0.167±0.006	7.6×10^−09^
TG (mM)	0.564±0.024	0.381±0.023	1.43×10^−06^
Cholesterol (mM)	3.376±0.012	3.431±0.012	0.0023
Cholesterol-HDL (mM)	0.752±0.019	0.78±0.024	ns

Plasma metabolites and hormones parameters of 7-mo-old male F1-CD and F1-LPD mice were measured in plasma of over-night starved animals and are expressed as means ± sem for n = 10 (Glucose) or n = 25 (Insulin, TG, Cholesterol, Cholesterol-HDL).

### F1-LPD mice are resistant to high fat diet-induced obesity

**Figure 3:**
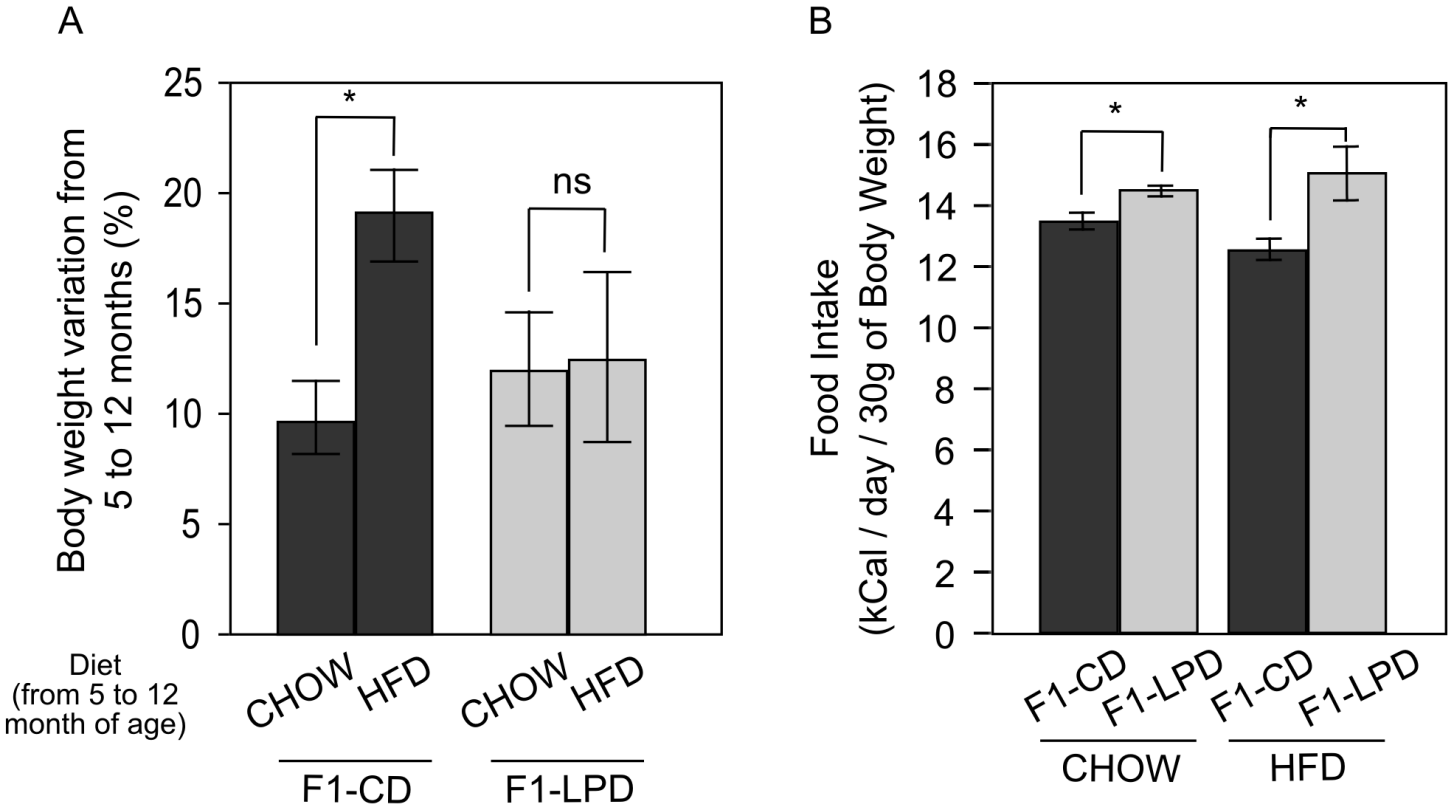
Weight gain and food intake of F1 mice subjected to HFD. Five-mo-old F1-CD and F1-LPD male mice were fed for 7 months with either a Chow diet or a HFD. A) Body weight variation was determined at the end of the diet challenge as a percentage of the initial body weight and is expressed as a mean ± sem for at least 8 mice/group. *p≤0.05. B) Food intake of 12-mo-old Chow or HFD-fed F1-CD and F1-LPD male mice was measured as described in Materials and Methods. Values represent the weight of food consumed daily (kCal) relatively to the weight of each mouse and are expressed as means ± sem for at least 8 mice/group. *p≤0.05.

According to the phenotype described above, one could hypothesize that F1-LPD mice may be resistant to High Fat Diet (HFD)-induced obesity. At the age of 5 months, F1-CD and F1-LPD males were fed either a Chow Diet (Chow) or HFD for 7 months. Figure 3A shows that HFD caused a significant increase in body weight of the F1-CD mice compared to Chow-fed animals. The same nutritional challenge did not increase the body weight gain of F1-LPD mice over the same period of time. These data show that F1-LPD mice are resistant to HFD-induced obesity. It is also noticeable that the higher food intake observed in Chow-fed F1-LPD compared to Chow-fed F1-CD mice is still measured in HFD-fed F1-LPD compared to HFD-fed F1-CD mice (Figure 3B). These results suggest that the resistance of F1-LPD mice to HFD-induced obesity is not due to a modification of food intake but rather to an adaptative increase in energy expenditure.

### Effects of perinatal malnutrition on mitochondrial function

We next hypothesized that the metabolic origin of the phenotype described above in F1-LPD mice could be due to adaptations in mitochondrial functions. In order to investigate this hypothesis, we measured by qRT-PCR the expression of several genes encoding proteins involved in the mitochondrial function in epididymal White Adipose Tissue (WAT), Brown Adipose Tissue (BAT), and *gastrocnemius* muscle from either F1-CD or F1-LPD 7-months-old adult males fed a Chow-Diet since weaning. These tissues were chosen on the basis of their importance regarding the regulation of energy expenditure.

**Figure 4:**
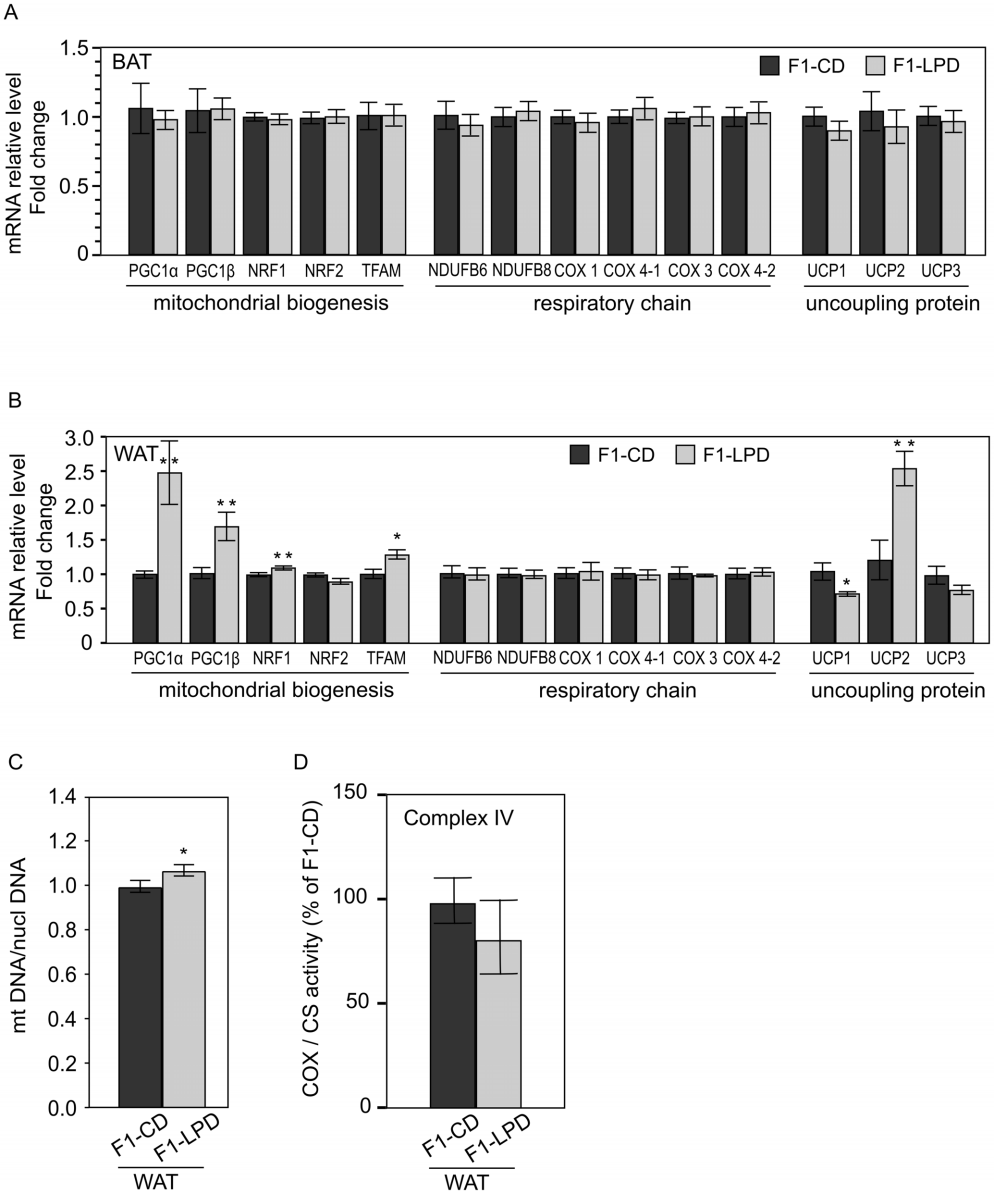
mRNA expression levels in Brown and White Adipose Tissue, Cytochrome C Oxidase and Citrate Synthase Activities and Mitochondrial DNA content in White Adipose Tissue of F1 mice. A, B) mRNA expression levels of genes encoding proteins involved in the mitochondrial function in (A) Brown Adipose Tissue (BAT) and (B) White Adipose Tissue (WAT) from 7-months-old F1-CD and F1-LPD males mice. mRNA relative levels are normalized relatively to Nono expression level and expressed as fold change relative to the control value measured in F1-CD. Values are means ± sem for at least 8 mice/group. **p≤0.05, *p≤0.08. C) Cytochrome C Oxidase (COX  =  Complex IV) and Citrate Synthase (CS) activities are measured in WAT from 7-months-old F1-CD and F1-LPD males mice and expressed as a percentage of the COX/CS ratio of F1-CD mice. Values are means ± sem for at least 8 mice/group. D) Mitochondrial density is estimated by measuring the mitochondria DNA (mtDNA) content relatively to the nuclear DNA (nuclDNA) content in White Adipose Tissue. mtDNA/nuclDNA ratio was calculated as the ratio of COX1 to cyclophilin A DNA levels, determined by real-time PCR, in DNA extracted from White Adipose Tissue of 7-months-old F1-CD and F1-LPD males mice. Results were normalized by the mean value of the control condition set to 1 unit. *p≤0.05.

Figure 4A shows that no modification was found in the expression of genes tested in BAT, and Figure 4B shows that, in WAT, none of the genes encoding respiratory subunits that have been measured presented any modification of expression. This is coherent with the lack of modification of COX/CS activity observed (Figure 4C). However in WAT, we found that PGC1α, PGC1β, NRF1 and TFAM gene expression are increased in F1-LPD mice (Figure 4B) suggesting that mitochondrial biogenesis may be affected. These results are coherent with the increased in mitochondrial DNA relative to nuclear DNA in the same tissue (Figure 4D). To strengthen this result, we performed, in WAT, a quantification of protein commonly used as markers of mitochondrial density like Porin protein level (by Western Blot) and citrate synthase (enzymatic activity). We found that F1-LPD mice tend to have both a higher expression of Porin (Figure S1A and B) and a higher Citrate synthase activity (Figure S2). However, even thought these differences failed to reach significativity, these results tend to confirm and are coherent with the overall results presented in Figures 4B and 4C. It is also noticeable that UCP2 gene expression (Figure 4B) and protein level (Figure S1A and B) was found to be strongly increased. This higher expression of UCP2 could play a role in the resistance to obesity as suggested by Fleury *et al*. [9]. Moreover the lack of expression of UCP1 in WAT suggests that no beige Adipose Tissue can be detected in the WAT of F1-LPD mice.

**Figure 5:**
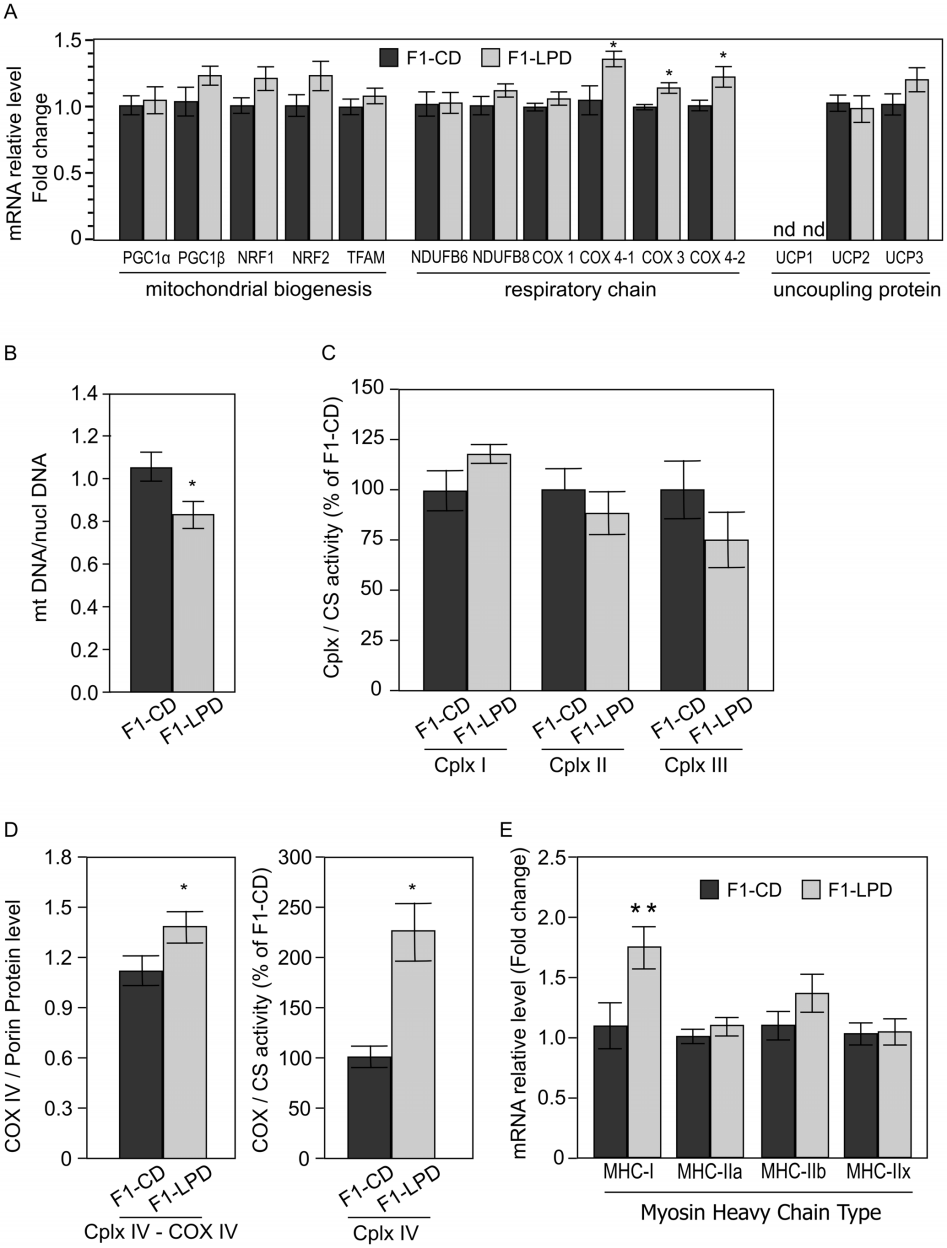
mRNA expression levels, Mitochondrial DNA content, Complex I, II and III activities, COX-IV protein content, Cytochrome C Oxidase and Citrate Synthase Activities and mRNA expression levels of Myosin Heavy Chain types in Gastrocnemius of F1 mice. A) mRNA expression levels of genes encoding proteins involved in the mitochondrial function in Gastrocnemius from 7-months-old F1-CD and F1-LPD males mice. mRNA relative levels are normalized relatively to HPRT1 expression level and expressed as fold change relative to the control value measured in F1-CD. Values are means ± sem for at least 8 mice/group. *p≤0.05. B) Mitochondrial density is estimated by measuring the mitochondria DNA (mtDNA) content relatively to the nuclear DNA (nuclDNA) content in Gastrocnemius from 7-months-old F1-CD and F1-LPD males mice. mtDNA/nuclDNA ratio was calculated as the ratio of COX1 to cyclophilin A DNA levels, determined by real-time PCR, in DNA extracted from gastrocnemius of 7-months-old F1-CD and F1-LPD males mice. Results were normalized by the mean value of the control condition set to 1 unit. *p≤0.05. C) Complex I, II and III and Citrate Synthase (CS) activities are measured in gastrocnemius from 7-months-old F1-CD and F1-LPD males mice and expressed as a percentage of the Cplx/CS ratio of F1-CD mice. Values are means ± sem for at least 8 mice/group. D) Left panel: Cytochrome C Oxidase Sub-Unit IV (COX-IV) protein level was assessed by western blotting in gastrocnemius from 7-months-old F1-CD and F1-LPD males mice. Histograms shown represent a densitometrical quantification after Porin normalization. Values are means ± sem for at least 4 mice/group. *p≤0.05. Right panel: Cytochrome C Oxidase (COX) and Citrate Synthase (CS) activities are measured in gastrocnemius from 7-months-old F1-CD and F1-LPD males mice and expressed as a percentage of the COX/CS ratio of F1-CD mice. Values are means ± sem for at least 8 mice/group. *p≤0.05. E) mRNA expression levels of the 4 different types of Myosin Heavy Chain (MHC-I, MHC-IIa, MHC-IIb, MHC-IIx) in Gastrocnemius from 7-months-old F1-CD and F1-LPD males mice. mRNA relative levels are normalized relatively to HPRT1 expression level and expressed as fold change relative to the control value measured in F1-CD. Values are means ± sem for at least 8 mice/group. *p≤0.05.

Thereafter, we chose to explore the *gastrocnemius* muscle for several reasons: firstly because it is a quantitatively important tissue and secondly because it is a mixed muscle, which metabolism can be considered as representative of the whole body musculature. In *gastrocnemius*, the mRNA expression of several of the tested COX subunit (Cytochrome-c Oxidase) is specifically induced in F1-LPD mice (Figure 5A), suggesting a modification of the expression of complex IV. To confirm this hypothesis, we measured the activity of the mitochondrial Complex I, II, III and IV in gastrocnemius extract from F1-CD and F1-LPD animals. Whereas the activity of the Complex I, II and III are not significantly affected by perinatal malnutrition (Figure 5C), we found that complex IV activity was indeed significantly increased in the F1-LPD muscle (Figure 5D, right panel). This result was confirmed by a western blot analysis against the sub-unit IV (COX-IV) of the complex IV and we found that, indeed, the protein level is increased in F1-LPD animals compared to F1-CD animals (Figure 5D-left panel and Figure S1C and D). Surprisingly, we found that mitochondrial DNA relative to nuclear DNA ratio shows a significant decrease in muscle mitochondrial content (Figure 5B) indicating a decrease in mitochondrial density in the muscle. To strengthen this result, we performed, in gactrocnemius, a quantification of protein commonly used as markers of mitochondrial density like Porin protein level (by Western Blot) and citrate synthase (enzymatic activity). We found that F1-LPD mice tend to have both a lower expression of Porin (Figure S1C and D) and a lower Citrate synthase activity (Figure S2). However, even thought these differences failed to reach significativity, these results tend to confirm the decrease in mitochondrial content shown in Figure 5B. Even thought his finding is striking, it could be hypothesized that it is related to an increased respiratory chain decoupling [10], [11] (i.e. to slip induction/trigger in COX) and to an enhanced thermogenesis [12]. Such an observation suggests that mitochondrial adaptations in F1-LPD muscle may be a cause of energy wasting. This has been rarely described in physiological situations [11], [12], but it is in agreement with the enhanced body temperature and minimal energy expenditure observed in F1-LPD mice compared to F1-CD.

To explore more extensively the hypothesis of an increase in muscle oxidative capacity, we quantified, the proportion of the different type of fibers in gastrocnemius of 7-months-old F1-CD and F1-LPD males. mRNA level of the 4 different types of Myosin Heavy Chain (MHC-I, MHC-IIa, MHC-IIb, MHC-IIx) was assessed by qRT-PCR. As shown Figure 5E, F1-LPD mice exhibit a significant increase in type I muscle fibers in the gastrocnemius muscle compared to F1-CD mice. Data from literature suggest that a greater proportion of type I fibers may be associated with a greater capacity for fat oxidation, which would favor resistance to body fat accumulation [13]. Overall, these data support the F1-LPD resistance against a HFD-induced obesity.

## Discussion

Metabolic diseases have multiple origins, including genetic and environmental factors and their patho-physiological characteristics are diverse and complex. Transient environmental influences, such as a nutritional stress during perinatal life, may have deleterious long lasting health effects that may last for the entire life of affected individuals [1], suggesting a fetal origin of metabolic diseases, a field that have been of growing interest in the last years. Moreover, the association of Type II diabetes with obesity and inactivity suggests an important link between energy homeostasis and the onset of metabolic disease [14]. Given the central role for mitochondria in energy metabolism, disordered mitochondrial function at the cellular level can impact whole-body metabolic homeostasis. Thus, the hypothesis that defective or insufficient mitochondrial function might play a potentially pathogenic role in mediating risk of T2DM and obesity has emerged in recent years.

It is of great interest to note that different experimental paradigms of perinatal exposure to under-nutrition or over-nutrition may be associated 1) with different phenotype in the adult offspring regarding energy expenditure, food intake and body weight 2) with different susceptibilities to various symptoms associated with the metabolic syndrome. For example, when female mice are subjected to under-nutrition only during gestation (and not during lactation), offspring have a lower birth weight and catch up quickly during early days of life (personal unpublished data and for review, see [15]). In the present study, however, we used a mouse model of adult offspring whose mothers have been fed a Low Protein Diet (F1-LPD) during gestation and lactation. We have previously shown that these animals have a lower body weight compared to the control and that they do not experience catch up growth even thought they increase their food intake [5]. However, it is important to note that results may vary depending on the specie (Rats vs mice) and on the exact nature of the diet. This may explain the apparent contradiction of our results with those of other publications showing an increase in visceral adipose tissue in a rat model of maternal protein restriction [16]. In the contrary, our results are coherent with epidemiological studies showing that two different famines produced different phenotype in the descendants depending on whether catch-up growth during early childhood occurred (Netherland cohort) or not (Leningrad cohort) as reviewed in [17].

Considering the blood parameters measured, our results are coherent with a resistance to the development of metabolic syndrome, as stated in the medical definition of this syndrome (Metabolic syndrome corresponds to elevated fasting plasma glucose, high serum triglycerides, low high-density cholesterol (HDL) levels). Importantly, we also showed that, once adult, these mice are protected against a high fat diet-induced obesity later in life. We thus investigated the hypothesis of an energy wasting process observed in these animals. We showed that the energy wasting process could be explained by an increase in energy expenditure in F1-LPD animals associated with a higher fat oxidation. The increased spontaneous activity related to foraging of the F1-LPD animals probably does not significantly participate to the increased energy expenditure because we also observed a significantly higher elevated body temperature and minimal energy expenditure that both suggest that an elevated resting metabolic rate is probably the main factor responsible for the elevated total energy expenditure and consequently to the resistance to an obesogenic diet. In that context, we showed that the increased energy expenditure is most likely due to specific mitochondrial adaptations in WAT and skeletal muscle, which potentially result in enhanced decoupling and thermogenesis. Altogether, our investigations showed that mitochondrial function is affected by perinatal undernutrition in WAT and muscle. Even thought the mRNA expression of none of the genes that has been tested was found to be modified in F1-LPD BAT, it would be interesting to further address, by using specific experiments, the role of this tissue in the phenotype of F1-LPD mice.

It has been shown that UCP2 have significant effects on obesity in mice, and that its mechanism of action may include alterations of lipid metabolism and metabolic rate [18]. In WAT, we observed a marked induction of UCP2 in F1-LPD animals, which can leads to two consequences. Firstly, a high UCP2 activity can prevent lipid storage by facilitating fatty acid oxidation. We indeed observed a reduced RQ reflecting a relatively higher rate of lipid oxidation in F1-LPD mice that can explain the low adiposity of these animals. Secondly, it could also be expected that, by uncoupling the oxidative phosphorylation (OXPHOS), the higher expression of UCP2 could participate to the observed increase in energy expenditure. Enhanced UCP2 gene and/or protein expression has been reported in several animal models of resistance to obesity [19]–[24]. These mechanisms are also supported by findings from Horvath et al [25] using mice moderately overexpressing UCP2.

In muscle, maternal under-nutrition differently affects mitochondrial function compared to what is observed in WAT. Strikingly, we observed a higher activity of the mitochondrial complex IV in F1-LPD compared to F1-CD. This finding was specific to complex IV since the maximal activity of the complexes I, II and III was similar between F1-LPD and F1-CD. It is of importance to note that Capaldi et al [26] reported that Complex IV is found in ≈5 fold excess over the other electron transport complexes of the mitochondria, which may indicate the physiological significance of excess complex IV [27]. This adaptation can be related to enhanced slipping processes within complex IV, which may be related to an energetic wasting process associated with an enhanced energy expenditure and an increased thermogenesis [12]. This observation is striking since mitochondrial density seems to be reduced in F1-LPD compared to F1-CD, thus suggesting that a unique adjustment of muscle mitochondrial function occurs in response to perinatal protein malnutrition. Such adaptations have been rarely described in physiological situations, but were reported in vitro for example in response to high doses of the local anesthetic bupivacaine [10], [11], or in response to long-term treatment with dexamethasone [28]. The increased mRNA expression of complex IV subunits reported in the present study implies a coordinated stimulation of transcription of both nuclear and mitochondrial genomes, but the molecular mechanisms involved in those effects are poorly understood. From *in vitro* approaches, Desquiret et al. [28] reported that the specific increase in complex IV enzymatic activity was mediated through the cytosolic glucocorticoid receptor in response to dexamethasone treatment, but the involvement of such a mechanism is not known to date in the context of maternal under-nutrition. Complex IV is the key regulator of the respiratory chain activity; it possesses a low capacity of reserve but exerts a large control on endogenous respiration rate [29]. Its increased activity in our study indicates a compensatory adaptation to uncoupled conditions and decreased oxidative phosphorylation efficiency. This is in agreement with the enhanced body temperature and minimal energy expenditure observed in F1-LPD mice compared to F1-CD. It also supports the F1-LPD resistance against a high fat diet-induced obesity. In the same way, our results regarding type I fiber increase in gastrocnemius of F1-LPD animals is coherent with the conclusion of Abou Mrad *et al*. stating that preexisting differences in muscle fiber composition may play a role in determining susceptibility to dietary obesity [13].

Our findings are consistent with the hypothesis that epigenetic modifications acquired during the early life may condition mitochondrial function in several tissues in later life. We have already shown that in the same nutritional model, the leptin gene regulation is affected by perinatal undernutrition via a modification in its promoter methylation. Further studies are now required to understand the epigenetic imprinting caused by perinatal nutrition on key genes regulating mitochondrial biogenesis and function. It has been previously demonstrated that mitochondrial function can be modified by a nutritional challenge. Indeed, changes in mitochondrial number and function in skeletal muscle and WAT in response to a high-fat-diet have been previously published [30]–[32]. Several studies revealed that mitochondria might be a target for fetal programming [33]–[37]. All these studies were conducted using a rat model of maternal malnutrition leading to a phenotype associated with catch up growth. For example, it was shown that malnutrition during the gestation period affects the mitochondrial function in pancreatic islets of the adult offspring leading to loss of pancreatic islet function and finally to insulin resistance [38]. In our mouse model of maternal undernutrition, however, we generate mice exhibiting an opposite phenotype (resistance to diet-induced-obesity, no insulin resistance). We indeed also observed a mitochondrial function modification in our model but this modification leads to a higher oxidative capacity in muscle, correlated with an increased energy expenditure, which is coherent with our phenotype. Clearly, different paradigms of exposure to under-nutrition or over-nutrition may be associated with different metabolisms of energy expenditure, food intake, weights and different susceptibilities to various symptoms associated with the metabolic syndrome. Taken together these results demonstrate that intra-uterine environment is a major contributor to the future of individuals and disturbance at a critical period of development may compromise their health. Consequently, understanding the molecular mechanisms may give access to useful knowledge regarding the onset of metabolic diseases.

## Supporting Information

Figure S1 **Western Blot and densitometrical quantification in Muscle and WAT for Porin, β-Actin, COX IV and UCP2.** A) Western Blot analysis of UCP2, Porin and β-Actin protein level in WAT from 7-months-old F1-CD and F1-LPD males mice. B) Densitometrical intensity ratio of Porin and UCP2 protein level normalized to β-Actin. Values are means ± sem for at least 4 mice/group. *p≤0.05. C) Western Blot analysis of COX-IV, Porin and β-Actin protein level in Gasctrocnemius from 7-months-old F1-CD and F1-LPD males mice. D) Densitometrical intensity ratio of Porin and COX-IV protein level normalized to β-Actin. Values are means ± sem for at least 4 mice/group. *p≤0.05.
(TIF)

Figure S2 **Citrate Synthase activity in muscle and WAT.** Citrate Synthase activity is measured in WAT and Gastrocnemius from 7-months-old F1-CD and F1-LPD males mice and expressed in mmol/min/mg. Values are means ± sem for at least 8 mice/group.
(TIF)

Table S1 **Sequences of mouse qPCR primers.** Sequences of mouse-specific primers used for qPCR analysis.
(PDF)
