# Supplementary figures and images for: Perinatal Protein Malnutrition Affects Mitochondrial Function in Adult and Results in a Resistance to High Fat Diet-Induced Obesity
Source: PLoS One. 2014 Aug 13;9(8):e104896. doi: 10.1371/journal.pone.0104896 (PMC4132016; doi:10.1371/journal.pone.0104896)

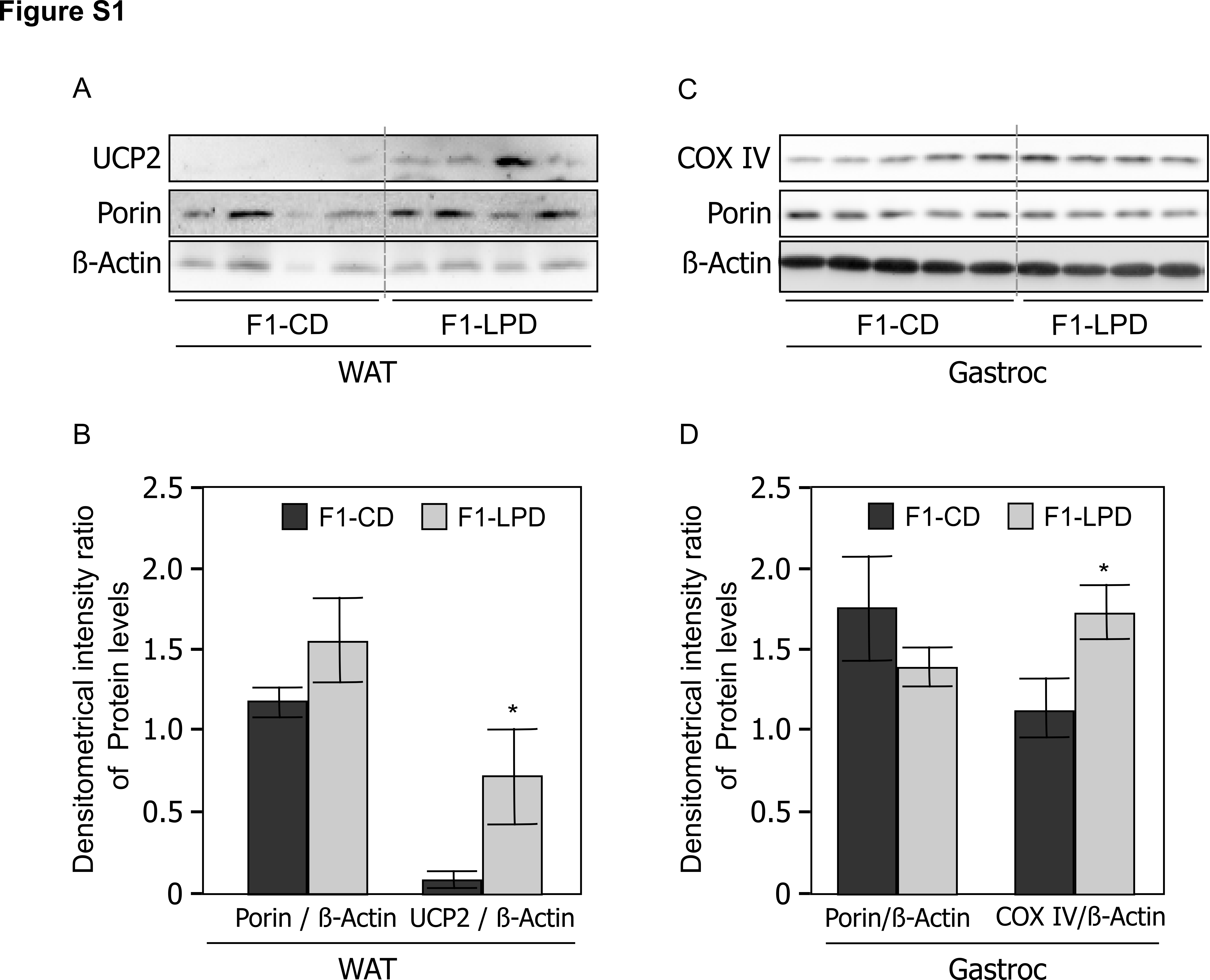

Supplement: Figure S1 — Western Blot and densitometrical quantification in Muscle and WAT for Porin, β-Actin, COX IV and UCP2. A) Western Blot analysis of UCP2, Porin and β-Actin protein level in WAT from 7-months-old F1-CD and F1-LPD males mice. B) Densitometrical intensity ratio of Porin and UCP2 protein level normalized to β-Actin. Values are means ± sem for at least 4 mice/group. *p≤0.05. C) Western Blot analysis of COX-IV, Porin and β-Actin protein level in Gasctrocnemius from 7-months-old F1-CD and F1-LPD males mice. D) Densitometrical intensity ratio of Porin and COX-IV protein level normalized to β-Actin. Values are means ± sem for at least 4 mice/group. *p≤0.05. (TIF) [file pone.0104896.s001.tif]

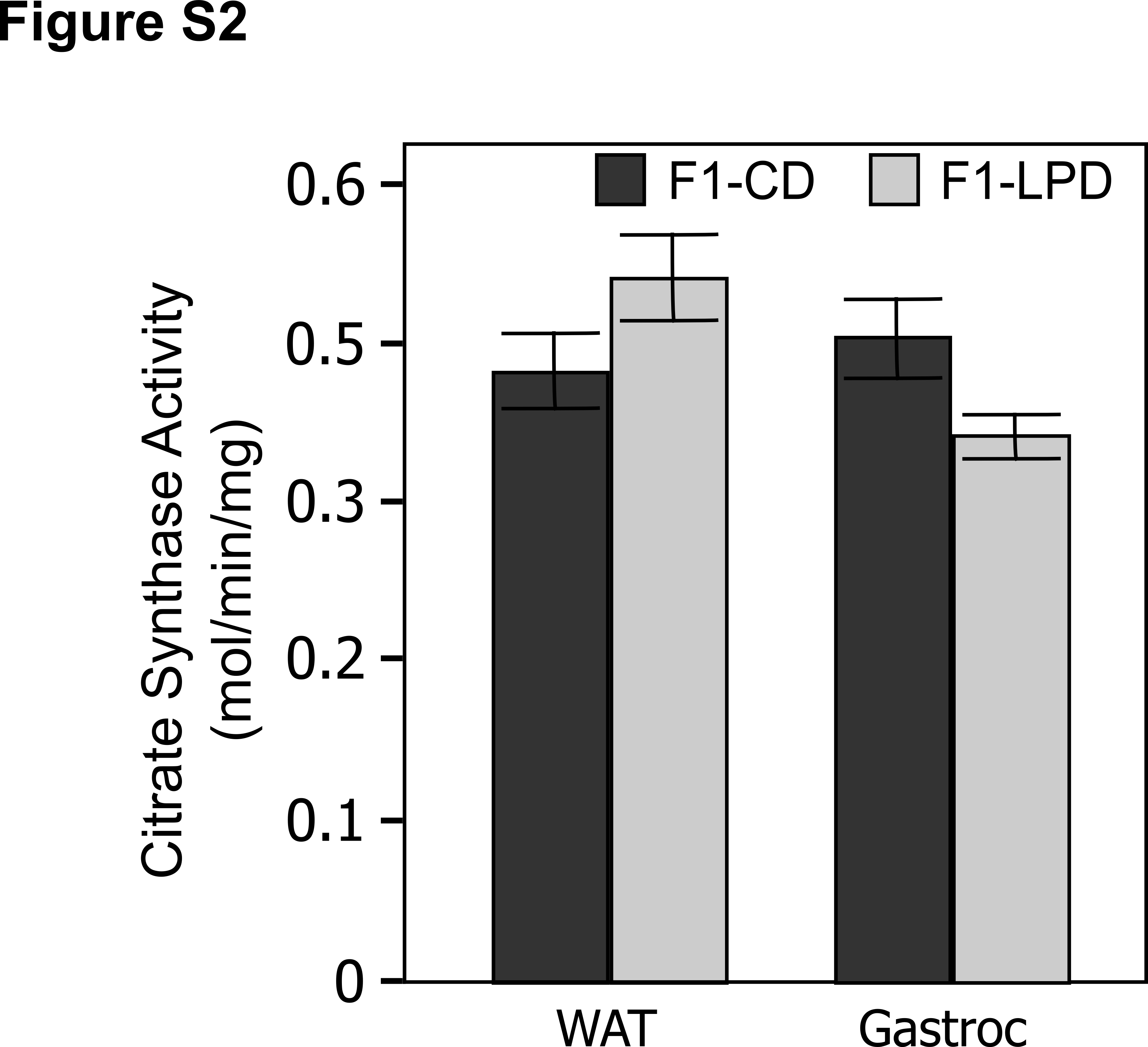

Supplement: Figure S2 — Citrate Synthase activity in muscle and WAT. Citrate Synthase activity is measured in WAT and Gastrocnemius from 7-months-old F1-CD and F1-LPD males mice and expressed in mmol/min/mg. Values are means ± sem for at least 8 mice/group. (TIF) [file pone.0104896.s002.tif]
