# Supplementary material for: Perinatal Protein Malnutrition Affects Mitochondrial Function in Adult and Results in a Resistance to High Fat Diet-Induced Obesity
Source: PLoS One. 2014 Aug 13;9(8):e104896. doi: 10.1371/journal.pone.0104896 (PMC4132016; doi:10.1371/journal.pone.0104896)
Supplement: Table S1 — Sequences of mouse qPCR primers. Sequences of mouse-specific primers used for qPCR analysis. (PDF) [file pone.0104896.s003.pdf]

TABLE S1: Sequences of mouse qPCR primers

COX1	Forward Primer	5'-TCGGAGCCCCAGATATAGCA -3'
	Reverse Primer	5'-TTTCCGGCTAGAGGTGGGTA -3'
COX3	Forward Primer	5'-CAAGGCCACCACACTCCTAT -3'
	Reverse Primer	5'-ATTCCTGTTGGAGGTCAGCA -3'
COX4-1	Forward Primer	5'-AGTGTTGTGAAGAGTGAAGAC -3'
	Reverse Primer	5'-GCGGTACAACTGAACTTTCTC -3'
COX4-2	Forward Primer	5'-CTGCCCGGAGTCTGGTAATG -3'
	Reverse Primer	5'-CAGTCAACGTAGGGGGTCATC -3'
NDUFB6	Forward Primer	5'-TCGCTGTTTCTCATGTGCTT -3'
	Reverse Primer	5'-TCTCCAGTCTCCAGAATTGTATCA -3'
NDUFB8	Forward Primer	5'-GGCCGCCAAGAAGTATAACA -3'
	Reverse Primer	5'-TGATACCACGGATCCCTCTC -3'
NRF1	Forward Primer	5'-CAGCACCTTTGGAGAATGTG -3'
	Reverse Primer	5'-CCTGGGTCATTTTGTCCACA -3'
NRF2	Forward Primer	5'-GATCCGCCAGCTACTCCCAGGTTG -3'
	Reverse Primer	5'-CAGGGCAAGCGACTCATGGTCATC -3'
PGC1-α	Forward Primer	5'-AGCCGTGACCACTGACAACGAG -3'
	Reverse Primer	5'-GCTGCATGGTTCTGAGTGCTAAG -3'
PGC1-β	Forward Primer	5'-TGGAAAGCCCCTGTGAGAGT -3'
	Reverse Primer	5'-TTGTATGGAGGTGTGGTGGG -3'
TFAM	Forward Primer	5'-CAAGTCAGCTGATGGGTATGG -3'
	Reverse Primer	5'-TTTCCCTGAGCCGAATCATCC -3'
UCP1	Forward Primer	5'-GGCATTCAGAGGCAAATCAGCT -3'
	Reverse Primer	5'-CAATGAACACTGCCACACCTC -3'
UCP2	Forward Primer	5'-TCTACAATGGGCTGGTCGC -3'
	Reverse Primer	5'-CAAGCGGAGAAAGGAAGGC -3'
UCP3	Forward Primer	5'-CCTACAGAACCATCGCCAGG -3'
	Reverse Primer	5'-ACCGGGGAGGCCACCACTGT -3'

HPRT1	Forward Primer<br>Reverse Primer	5'-TCCTCCTCAGACCGCTTTT-3'<br>5'-CCTGGTTCATCATCGCTAATC-3'
NONO	Forward Primer<br>Reverse Primer	5'-TGCTCCTGTGCCACCTGGTACTC-3'<br>5'-CCGGAGCTGGACGGTTGAATGC-3'
MHC-I	Forward Primer<br>Reverse Primer	5'-CTCCCAAGGAGAGACGACTG-3'<br>5'-TTAAGCAGGTCGGCTGAGTT-3'
MHC-IIa	Forward Primer<br>Reverse Primer	5'-AAAGCTCCAAGGACCCTCTT-3'<br>5'-AGCTCATGACTGCTGAACTCAC-3'
MHC-IIb	Forward Primer<br>Reverse Primer	5'-TGGCCGAGCAAGAGCTAC-3'<br>5'-TTGATGAGGCTGGTGTTCTG-3'
MHC-IIx	Forward Primer<br>Reverse Primer	5'-AATCAAAGGTCAAGGCCTACAA-3'<br>5'-GAATTTGGCCAGGTTGACAT-3'
